# An Interactive Web Portal for Tracking Oncology Patient Physical Activity and Symptoms: Prospective Cohort Study

**DOI:** 10.2196/11978

**Published:** 2018-12-21

**Authors:** Michael Marthick, Haryana M Dhillon, Jennifer A Alison, Bobby S Cheema, Tim Shaw

**Affiliations:** 1 Chris O'Brien Lifehouse Camperdown Australia; 2 Centre for Medical Psychology & Evidence-based Decision-making School of Psychology University of Sydney Sydney Australia; 3 Faculty of Health Sciences University of Sydney Sydney Australia; 4 Sydney Local Health District Sydney Australia; 5 School of Science and Health Western Sydney University Penrith Australia; 6 Research in Implementation Science and eHealth Group Faculty of Health Sciences University of Sydney Sydney Australia

**Keywords:** physical activity, fitness trackers, eHealth, neoplasms

## Abstract

**Background:**

Physical activity levels typically decline during cancer treatment and often do not return to prediagnosis or minimum recommended levels. Interventions to promote physical activity are needed. Support through the use of digital health tools may be helpful in this situation.

**Objective:**

The goal of the research was to evaluate the feasibility, usability, and acceptability of an interactive Web portal developed to support patients with cancer to increase daily physical activity levels.

**Methods:**

A Web portal for supportive cancer care which was developed to act as a patient-clinician information and coaching tool focused on integrating wearable device data and remote symptom reporting. Patients currently receiving or who had completed intensive anticancer therapy were recruited to 3 cohorts. All cohorts were given access to the Web portal and an activity monitor over a 10-week period. Cohort 2 received additional summative messaging, and cohort 3 received personalized coaching messaging. Qualitative semistructured interviews were completed following the intervention. The primary outcome was feasibility of the use of the portal assessed as both the number of log-ins to the portal to record symptoms and the completion of post-program questionnaires.

**Results:**

Of the 49 people were recruited, 40 completed the intervention. Engagement increased with more health professional contact and was highest in cohort 3. The intervention was found to be acceptable by participants.

**Conclusions:**

The portal was feasible for use by people with a history of cancer. Further research is needed to determine optimal coaching methods.

## Introduction

Physical activity levels typically decline during cancer treatments such as chemotherapy or radiation therapy and often fail to return to prediagnosis or minimum recommended levels [1]. Patients report symptoms and side effects, primarily cancer-related fatigue, as a significant barrier to increasing physical activity levels [2-3]. These factors present a challenge for health professionals to increase physical activity levels in cancer populations. Integrating physical activity and exercise prescription into routine clinical care is supported by various national and international statements and guidelines that emphasize the importance of contact with an exercise professional with expertise in cancer care [4-5].

In order to promote physical activity, health professionals, particularly exercise professionals, may suggest the use of commercially available physical activity trackers to their patients. However, data monitoring by health professionals for a large number of patients who use such trackers can be difficult because individual patient data is not readily available as it sits with the patient.

Digital health interventions such as the use of Web portals have been shown to be beneficial by supporting engagement in health and wellness activities in individuals with chronic diseases, including people with a history of cancer. A Web portal is generally seen as a secure website that brings information from various sources together in a uniform way [6-7]. Web portals can have many uses including patient access to personal medical records, appointments, medications, communication with health professionals, and decision support tools [8-12].

Patients who use Web portals may have greater engagement in their treatment, increased treatment satisfaction, and better communication with their health professional care team. This includes a method to record and track patient-reported outcome (PRO) measures [6-9,13]. These factors may contribute to facilitating positive health behavior change.

The use of an integrated clinician-patient Web portal, with a mechanism for automated real-time data transfer, may provide the ability to track physical activity and patient-reported symptoms such as fatigue and provide an opportunity to positively impact behavior change through messaging. The use of messaging, including personalized coaching messaging, has emerged as a promising approach to promoting positive behavior change [10,11]. However, the feasibility of Web portals to support physical activity behavior change in people with a history of cancer has not been evaluated.

The aim of the study was to assess the feasibility of using a Web portal with activity monitoring and personalized messaging for people diagnosed with and treated for cancer.

## Methods

### Study Design

This was a prospective, longitudinal cohort study to determine user feasibility of a Web portal in cancer patients. The study protocol has been published previously [12].

### Web Portal

An interactive Web portal was developed that included integration of real-time wearable activity device data, collection of PROs and symptom information, the provision of educational material, and individualized coaching messaging to support behavior change by encouraging patient engagement in physical activity. The Web portal enabled remote monitoring of physical activity for use by both clinician and patient, along with symptom and health-related quality of life (HRQoL) tracking capabilities. The Web portal also allowed for educational emails, summary messaging, and individual personalized messages to be sent to participants.

### Activity and Sleep Tracker

The Misfit Shine activity monitor was used in this study. The Shine was chosen due to its long battery life. Participants enrolled in the study could also opt to bring their own device from the Misfit, Garmin, or Fitbit product ranges.

### Study Population

The inclusion criteria were (1) diagnosed with any cancer, at any stage of treatment receiving or had received anticancer therapy within the last 12 months, (2) aged 18 years or older, (3) Eastern Cooperative Oncology Group performance status 0 to 2, (4) had internet or mobile phone access, (5) willing to complete the intervention and follow-up in English, and (6) provided written informed consent. Participants were excluded if they were unable or had limited ability to speak English or had any condition that would compromise their ability to understand the participant information or give informed consent.

### Recruitment

Potentially eligible patients registered with the cancer center were invited to participate by a member of their health care team between March and June 2017. Following eligibility check and consent, participants were enrolled serially into one of three cohorts without randomization. Cohort 1 was provided Web portal access and given a wearable activity tracker (Misfit Shine) for 10 weeks. Cohort 2 was provided Web portal access, an activity tracker, and an additional weekly automated summary message via the Web portal detailing average symptom and physical activity scores over the past week, along with specific educational material such as information on cancer-related fatigue and nutrition. Cohort 3 received the same content as Cohort 2 plus personalized behavioral change messaging from an accredited exercise physiologist (MM). Messages were sent weekly through the Web portal to the participants’ email. Each participant received a 20- to 30-minute face-to-face onboarding and setup session.

### Primary Outcome

The primary outcome was the feasibility of the program. The intervention was deemed feasible if a compliance rate of more than 70% was observed. Compliance comprised two measures:

- Log-ins: a patient was defined as compliant if they had more than 2 log-ins to record symptoms over the 10-week study period
- Questionnaires: a patient was defined as compliant if they completed the follow-up questionnaire at week 10.

For the Web portal to be deemed feasible, more than 70% of the participants needed to comply with both criteria.

### Secondary Outcomes

The secondary objectives of the study were to describe the number of individuals who were eligible, took up the program, and completed the program; compute the rate of goal attainment as set by the exercise physiologist in week 2; and measure participant satisfaction, acceptability with the intervention, self-efficacy related to change in lifestyle factors, and changes in PROs including symptom and HRQoL scores. For cohorts 2 and 3, median daily step count was recorded and weekly email engagement measured.

At the initial face-to-face session, baseline PROs were completed on the Web portal, with follow-up questionnaires administered remotely. Three validated PRO measures were used: symptom tracking scale—Edmonton Symptom Assessment Scale (ESAS) [14], HRQoL tool—Functional Assessment of Cancer Therapy–General (FACT-G) [15], and self-efficacy scale— Cancer Behavior Inventory–Brief Version (CBI-B) [16]. An additional study-specific feedback questionnaire was remotely administered via a Web survey to assess participant satisfaction with the intervention, focused on the Web portal and activity tracker.

### Data Analysis

Baseline demographics are summarized as number and percentage for categorical variables and mean and standard deviation or median and interquartile range (IQR) for continuous variables depending on the distribution. The number of compliant participants within each cohort is summarized as number and percentage. The number and percentage of patients who attained their step goal was summarized weekly and by cohort, along with the median number of weeks taken to attain goals. Daily step count was summarized at weeks 1 and 10 for each cohort as mean and standard deviation or median and IQR. The mean difference and 95% confidence interval for physical activity between weeks 1 and 10 is provided. HRQoL scores are summarized as median and IQR or mean and standard deviation at the initial and week 10 visit for each cohort group. The mean difference between time points is displayed alongside the 95% confidence interval. The number and percentage of opened emails is summarized for cohorts 2 and 3 by each week of the study, and the number of personalized messages opened by cohort 3 is summarized as number and percentage. The number of symptoms reported was used to investigate the association between baseline characteristics and engagement with the Web portal. A Mann-Whitney *U* test was used to compare the number of symptoms between categorical variables.

Participants were invited to complete a semistructured qualitative interview after completing the study in order to provide feedback regarding their perception of the acceptability of the intervention and experience in using the Web portal. Interviews were conducted by an experienced qualitative researcher (AJ) via telephone and audio recordings. Interviews were transcribed verbatim and analyzed thematically [17] using a framework approach [18]. Three coders (MM, JA, HMD) coded the data independently. Qualitative data were used to augment quantitative findings in this paper.

Permission to conduct this study was granted by the Royal Prince Alfred Hospital Human Research and Ethics Committee (X16-0051). All participants provided written informed consent.

## Results

### Participant Characteristics

**Figure 1:**
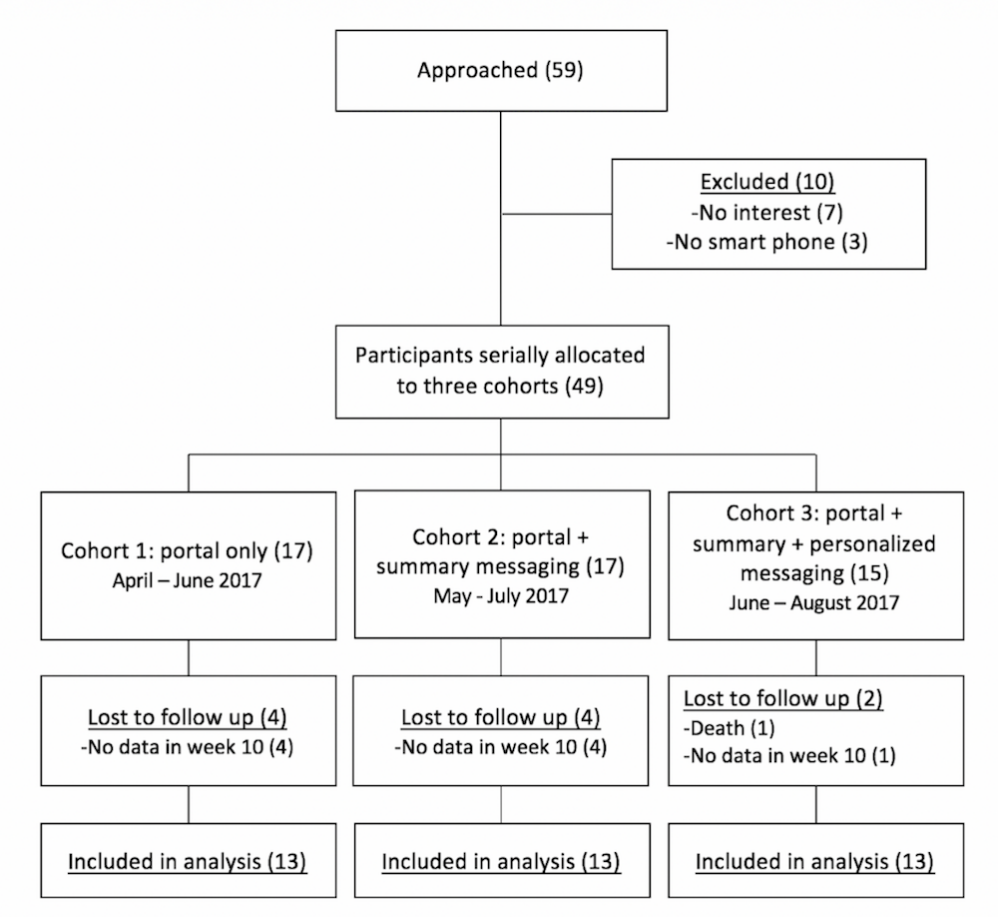
Study flow chart.

A total of 59 patients were invited to participate, and 83% of those (49/59) were recruited to the study. The first 17 participants were entered into cohort 1 in month 1, the second 17 into cohort 2 in month 2, and final 15 into cohort 3 in month 3. Recruitment numbers for each cohort were lower than planned (n=20) due to delay in the Web portal development, and 80% (39/49) of participants had data included in the analysis. There were no data predictive of patients lost to follow-up. Figure 1 shows the study flow chart.

Participants were mostly female (38/49, 78%) with a history of breast cancer (27/49, 55%), the median age was 54 years, and 24% (12/49) were receiving concurrent chemotherapy. Median time since last intensive anticancer therapy was 3.5 (IQR 0-12.5) months. The majority (43/49, 88%) had at least one comorbidity. Most participants (33/49, 67%) had not used an activity monitor previously, and the majority (40/49, 82%) were supplied with an activity monitor by the study investigators. Patient demographics are summarized in Table 1.

### Primary Outcome

#### Feasibility Measures

The number of log-ins and completed questionnaires are shown in Table 2. Feasibility increased across the cohorts, with cohort 1 having the least number of participants (7/17, 35%) and cohort 3 having the most (12/14, 86%) meeting the two criteria for feasibility. Feasibility criteria were met for cohort 3 only.

#### Participant Acceptability of Intervention

Twelve themes were identified from the data, with 4 themes directly applicable to the feasibility and acceptability of the intervention. Participants in the study generally reported a high level of acceptability for the intervention.

It was really, really positive and it was really helpful in terms of making a progressive recovery.

Participant 4, cohort 2

I think from both mental and physical point of view it was really worthwhile for me. 

Participant 1, cohort 3

### Secondary Outcomes

#### Symptom Logging

**Figure 2:**
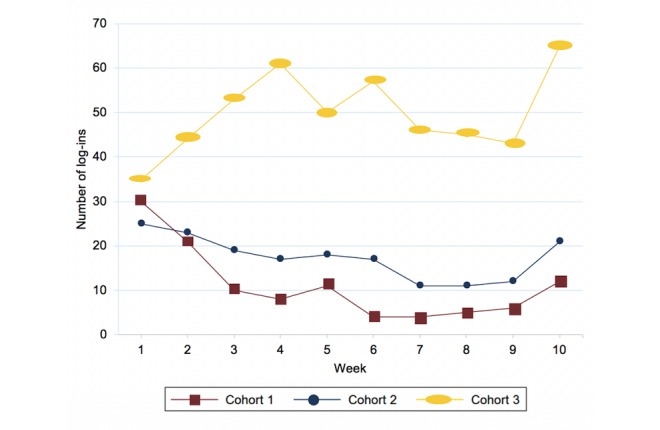
Mean log-ins to record symptoms per week for each cohort.

The mean number of log-ins to report symptoms increased in each of the cohorts depending on the level of interaction. Cohort 1 had a mean of 11 log-ins (range 0-52), cohort 2 had a mean of 17 log-ins (range 0-104) and cohort 3 had a mean of 50 log-ins (range 3-121). Figure 2 shows the number of log-ins to record symptoms per week across cohorts.

**1:** Summary of participant baseline characteristics by cohort.

Characteristics		Cohort 1 (n=17)	Cohort 2 (n=17)	Cohort 3 (n=15)	Total (N=49)
Age in years, mean (SD)		56 (10.0)	51 (10.9)	56 (12.4)	54 (11.0)
Gender, male, n (%)		2 (12)	4 (24)	5 (33)	11 (22)
**Diagnosis, n (%)**					
	Hematological	1 (6)	2 (12)	5 (33)	8 (16)
	Breast	12 (71)	12 (71)	3 (20)	27 (55)
	Prostate	1 (6)	1 (6)	1 (7)	3 (6)
	Colorectal	2 (12)	—^a^	1 (7)	3 (6)
	Lung	—	—	2 (13)	2 (4)
	Other	1 (6)	2 (12)	3 (21)	6 (12)
Current chemotherapy, n (%)		3 (18)	5 (29)	4 (27)	12 (24)
**Treatment history, n (%)**					
	Chemotherapy	11 (65)	7 (41)	13 (87)	—
	Radiation therapy	11 (65)	5 (29)	2 (30)	—
	Recent surgery (<6 weeks)	16 (94)	10 (59)	8 (53)	—
	Current hormonal therapy	7 (41)	7 (41)	4 (27)	—
	Immunotherapy	1 (6)	1 (6)	1 (7)	—
Time since last treatment, months, median (range)		5.95 (0.1-13.5)	1.38 (0-13.1)	2.17 (0-12.3)	3.5 (0-12.1)
**Comorbidities, number**					
	0	2 (12)	2 (12)	2 (13)	6 (12)
	1	4 (24)	8 (47)	7 (47)	19 (39)
	2	7 (41)	4 (24)	4 (27)	15 (31)
	3	4 (24)	3 (18)	2 (13)	9 (18)
Travel time to cancer center, minutes, median (range)		24 (5-45)	35 (10-120)	37 (5-240)	32 (5-240)
Previous use of activity tracker, n (%)		4 (24)	7 (41)	5 (33)	16 (33)
**Activity tracker**, n (%)					
	Supplied with Misfit Shine	15 (88)	12 (71)	13 (87)	40 (82)
	Using own Garmin or Fitbit	2 (12)	5 (29)	2 (13)	9 (18)

^a^Not applicable.

**Table 2:** Feasibility of study intervention.

Characteristics	Cohort 1 (n=17), n (%)	Cohort 2 (n=17), n (%)	Cohort 3 (n=15), n (%)
Log-ins, Web portal data logs (>2)	7 (41)	11 (65)	15 (100)
Questionnaires, completed at follow-up	12 (71)	11 (65)	12/14^a^ (86)
Log-ins and questionnaires combined	6 (35)	11 (65)	12/14^a^ (86)

^a^One patient death reported during study period.

**Figure 3:**
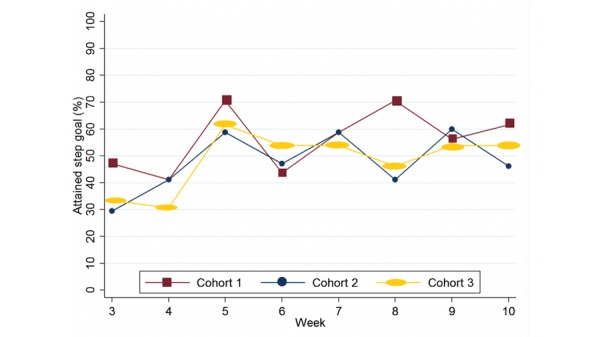
Proportion of participants who attained their step goal.

#### Goal Attainment

The number of patients who attained their daily step count goal are summarized for each week of the study in Figure 3. Week 1 and 2 of the intervention were used to work out an attainable goal, therefore data for weeks 3 to 10 are shown. A number of participants in each cohort had no activity tracker data as the weeks progressed, suggesting the activity tracker was no longer in use. At week 10 of the intervention, we received activity tracker data from 76% (13/17) of participants in cohort 1, 76% (13/17) of participants in cohort 2, and 93% (13/14) of participants in cohort 3, noting one participant died in cohort 3.

#### Activity Tracker Data

Daily step count has been summarized at week 1 and week 10 for each cohort in Table 3.

#### Acceptability of Device

The activity tracker given to participants (Misfit Shine) was generally well received, with participants stating they liked it and found it acceptable to use.

Yes. I absolutely love the thing that you wear on your arm. I'm just elated. I think it’s really motivating and I really enjoyed having that.

Participant 3, cohort 1

Absolutely, absolutely I loved it. It was really good to see exactly what it took to get to my goal each day and I love it. To the point I’m going to get another one and it’s going to be a part of my life to have a fitness tracker now.

Participant 3, cohort 3

Some participants reported concerns about the accuracy of the device, in particular sleep tracking.

So, I don’t think the [sleep] data is accurate, so I didn’t bother.

Participant 3, cohort 2

#### Patient-Reported Outcome Questionnaires

Changes in PRO questionnaire results from the initial intake (week 1) to end of program (week 10) are summarized below for each of the 3 cohort groups.

The ESAS was used to report pre-post symptom changes such as fatigue and pain and is reported in Table 4. A lower ESAS score indicates a lower symptom burden.

Change in patient-reported self-efficacy was reported using the CBI-B. A lower CBI-B score reflects improved self-efficacy. At a 95% confidence interval, cohort 1 had a change in score of –0.33 (–15.2 to 14.6), cohort 2 had a change of –6.78 (–21.9 to 8.3), and cohort 3 had a change of –2.18 (–11.9 to 7.6).

Change in patient-reported HRQoL was reported using the FACT-G and reported in Table 5. Lower scores on FACT-G indicate better HRQoL across 4 domains.

#### Weekly Email Learning Engagement

The most accessed educational topic was sleep (week 6) with 95% (16/17) and 93% (13/14) of participants in cohorts 2 and 3 opening the email, respectively, followed by nutrition (week 3) with 80% (14/17) and 93% (14/15) in cohorts 2 and 3, respectively, opening the email (see Table 6). A majority of participants in both groups engaged with educational content each week.

#### Practitioner Weekly Time

Participants in cohorts 1 and 2 received no direct health professional contact following onboarding. Cohort 3 received weekly personalized coaching messaging for which time data were collected. Weekly, the mean time spent by the health professional to interact with each participant was 11 minutes.

#### Acceptability of Web Portal Educational Content

Cohorts 2 and 3 had access to a curated selection of Web portal educational information including written and video content. The participants perceived the portal educational content to be acceptable.

I thought it was really good, the information was presented in a glaring manner.

Participant 4, cohort 2

Some respondents reported they would have preferred tailoring of content to their stage of cancer treatment and care.

Some of the stuff I might have been interested in two and a half years ago, but it’s not so relevant to me now.

Participant 5, cohort 3

#### Acceptability of Personalized Messaging

Qualitative interview data for those participants in cohort 3 who received personalized messaging revealed it was acceptable and provided additional motivation to help them use the Web portal and attain goal.

And it actually made me happy. It gave me a sense of achievement, especially when the [exercise physiologist] would send the message saying, “Wow, you've matched your goals. Well done.” I felt a lot of pride in myself.

Participant 1, cohort 3

...it made me just push myself and even on days when I didn't want to walk I thought no my steps were down and I should get out there and go for a walk and so on.

Participant 3, cohort 3

Reflecting on the lack of interaction, some participants in cohorts 1 and 2 remarked they would have liked more contact with their health professional throughout the study.

...but if someone motivated me to say, “Would you like to come in and have a look at that app again and I'll show you what it does. And let's see how you're going with it,” then that might have...I might have engaged with it a bit more...or at all.

Participant 3, cohort 1

**Table 3:** Secondary outcome: change in daily step count.

Daily step count	Cohort 1, median (IQR)^a^	Cohort 2, median (IQR)	Cohort 3, median (IQR)
Week 1	7549 (4835-10,138)	7193 (4206-9998)	6862 (4980-9202)
Week 10	8889 (6545-11,358)	7762 (5566-11,311)	8579 (6060-11,008)

^a^IQR: interquartile range.

**Table 4:** Secondary outcome: change in patient-reported symptom scores (Edmonton Symptom Assessment Scale).

Symptom	Cohort 1			Cohort 2			Cohort 3		
	Week 1 (n=17), median (IQR)^a^	Week 10 (n=11), median (IQR)	Change, median (IQR)	Week 1 (n=17), median (IQR)	Week 10 (n=11), median (IQR)	Change, median (IQR)	Week 1 (n=15), median (IQR)	Week 10 (n=11), median (IQR)	Change, median (IQR)
Pain	2 (0 to 3)	4 (1 to 5)	0 (0 to 1)	2 (0 to 3)	0 (0 to 2)	0 (–3 to 0)	2 (0 to 5)	1 (0 to 1)	–1 (–2 to 0)
Fatigue	4 (2 to 5)	5 (2 to 7)	0 (–1 to 3)	4 (3 to 5)	3 (2 to 4)	–1 (–2 to 2)	4 (0 to 5)	1 (1 to 2)	0 (–4 to 1)
Nausea	0 (0 to 0)	1 (0 to 3)	1 (0 to 3)	0 (0 to 2)	0 (0 to 0)	0 (0 to 0)	0 (0 to 0)	0 (0 to 0)	0 (0 to 0)
Depression	0 (0 to 1)	1 (0 to 3)	0 (0 to 2)	0 (0 to 3)	1 (0 to 4)	0 (0 to 1)	0 (0 to 1)	0 (0 to 1)	0 (0 to 0)
Anxiety	1 (0 to 2)	1 (0 to 3)	0 (–1 to 0)	0 (0 to 3)	1 (0 to 3)	1 (0 to 2)	0 (0 to 3)	0 (0 to 0)	0 (–3 to 0)
Drowsiness	2 (0 to 3)	2 (0 to 5)	3 (0 to 4)	0 (0 to 3)	0 (0 to 4)	0 (–3 to 1)	0 (0 to 4)	1 (0 to 3)	0 (–1 to 2)
Shortness of breath	0 (0 to 2)	0 (0 to 3)	0 (–1 to 1)	0 (0 to 0)	0 (0 to 0)	0 (0 to 0)	0 (0 to 3)	0 (0 to 2)	0 (–2 to 1)
Appetite	0 (0 to 2)	0 (0 to 4)	1 (0 to 2)	3 (0 to 5)	2 (0 to 4)	0 (–3 to 1)	1 (0 to 3)	0 (0 to 4)	0 (–1 to 0)
Sleep	5 (3 to 5)	2 (0 to 5)	0 (–3 to 0)	5 (3 to 6)	4 (3 to 5)	0 (–2 to 0)	5 (1 to 8)	2 (1 to 4)	–1 (–3 to 1)
Feeling of wellbeing	4 (2 to 5)	3 (3 to 4)	0 (–1 to 1)	4 (3 to 5)	3 (3 to 6)	–1 (–2 to 1)	3 (2 to 5)	2 (1 to 3)	0 (–1 to 1)
Financial distress	0 (0 to 2)	0 (0 to 2)	0 (–1 to 1)	1 (0 to 5)	1 (0 to 5)	0 (–2 to 1)	0 (0 to 3)	0 (0 to 2)	0 (0 to 0)
Spiritual pain	0 (0 to 0)	0 (0 to 3)	0 (0 to 1)	0 (0 to 1)	0 (0 to 1)	0 (–1 to 0)	0 (0 to 2)	0 (0 to 1)	0 (0 to 0)
Sadness	2 (0 to 2)	2 (1 to 4)	1 (0 to 2)	1 (0 to 2)	1 (0 to 6)	0 (0 to 2)	0 (0 to 3)	0 (0 to 1)	0 (–2 to 0)
Vomiting	0 (0 to 0)	0 (0 to 0)	0 (0 to 0)	0 (0 to 0)	0 (0 to 0)	0 (0 to 0)	0 (0 to 0)	0 (0 to 0)	0 (0 to 0)
Numbness/tingling	3 (1 to 3)	3 (3 to 4)	0 (–1 to 1)	0 (0 to 1)	0 (0 to 2)	0 (0 to 1)	1 (0 to 3)	1 (0 to 4)	–1 (–1 to 0)
Dry mouth	0 (0 to 2)	0 (0 to 2)	0 (0 to 0)	0 (0 to 2)	0 (0 to 0)	0 (–1 to 0)	0 (0 to 4)	0 (0 to 1)	0 (–2 to 0)
Memory	4 (3 to 5)	5 (4 to 6)	0 (0 to 2)	4 (2 to 5)	4 (2 to 5)	0 (–1 to 1)	2 (1 to 3)	1 (0 to 2)	0 (–1 to 1)
Distress	0 (0 to 3)	0 (0 to 2)	0 (–2 to 0)	0 (0 to 1)	2 (0 to 3)	2 (0 to 3)	0 (0 to 1)	0 (0 to 0)	0 (0 to 0)

^a^IQR: interquartile range.

**Table 5:** Secondary outcome: change in patient-reported quality of life (Functional Assessment of Cancer Therapy–General).

Domain	Cohort 1			Cohort 2			Cohort 3		
	Week 1 (n=17), median (IQR)^a^	Week 10 (n=17), median (IQR)	Change, median (IQR)	Week 1 (n=17), median (IQR)	Week 10 (n=17), median (IQR)	Change, median (IQR)	Week 1 (n=15), median (IQR)	Week 10 (n=15), median (IQR)	Change
Physical	7 (5 to 10)	2 (0 to 8)	–2 (–8 to 0)	7 (5 to 12)	0 (0 to 4)	–4 (–10 to 0)	4 (2 to 11)	3 (0 to 10)	0 (–4 to 0)
Social	22 (16 to 24)	11 (0 to 22)	–9 (–17 to 0)	21 (15 to 23)	0 (0 to 18)	–10 (–22 to –3)	22 (16 to 24)	15 (0 to 21)	–3 (–11 to 0)
Emotional	7 (5 to 8)	6 (0 to 9)	0 (–6 to 1)	6 (5 to 10)	0 (0 to 6)	–3 (–7 to 1)	5 (5 to 11)	6 (0 to 9)	0 (–5 to 1)
Functional	17 (13 to 20)	12 (0 to 16)	–8 (–14 to 0)	14 (12 to 17)	0 (0 to 16)	–4 (–13 to 1)	16 (7 to 18)	14 (0 to 19)	0 (–7 to 2)

^a^IQR: interquartile range.

**Table 6:** Secondary outcome: percentage of emails opened.

Week and topic	Cohort 2 (n=17), n (%)	Cohort 3 (n=15)^a^, n (%)
Week 1: Introduction	14 (82)	11 (73)
Week 2: Fatigue	13 (76)	12 (80)
Week 3: Nutrition	14 (82)	14 (93)
Week 4: Exercise	10 (59)	13 (87)
Week 5: Emotional health	12 (71)	11 (79)^a^
Week 6: Sleep	16 (94)	13 (93)^a^
Week 7: Pain	11 (65)	12 (86)^a^
Week 8: Qi Gong	14 (82)	11 (79)^a^
Week 9: Finances	12 (71)	13 (93)^a^
Week 10: Completion	11 (65)	11 (79)^a^

^a^After week 4, n=14 due to patient death during study period.

#### Participant Survey Feedback

Overall, the participant satisfaction with the intervention was high, with 83% (33/40) of respondents extremely satisfied or moderately satisfied with the intervention. Satisfaction with the activity tracker (Misfit Shine) was high, with 77% (31/40) of respondents extremely or moderately satisfied with using the device. In addition, 73% (29/40) of respondents found the Web portal extremely or moderately easy to use.

## Discussion

### Principal Findings

The results of this study demonstrate the feasibility of using a remote digital health intervention to track and promote physical activity levels and function and that personalized coaching messaging appears to increase participant engagement. The Web portal was found to be acceptable by the majority of participants, and satisfaction with its use was high across all cohorts. Participants accessing the Web portal varied widely in engagement, but overall participants in cohorts 2 and 3 interacted more with the Web portal compared to cohort 1.

The attrition rate was lowest in cohort 3, which had personalized contact, and highest in cohort 1, which had no personalized contact. This suggests simply giving patients access to a tool such as the Web portal was not sufficient to keep patients engaged for more than a short time. Regular interaction between patients and health professionals such as that provided by personalized messaging may lead to increases in participant accountability and may be a key method to improving engagement. Personalized messages are seen to be most effective when tailored to each patient rather than generalized to broader audiences [10,11,19].

The impact of messaging on engagement of participants was clear from the qualitative responses, where they were stated to be motivational and helpful. Further focus on the frequency, length, and content of personalized messaging will be an important development area for the future.

This research also builds on previous studies, such as those conducted by Huh et al [20] and Rosenberg et al [21], which indicated that patients support the idea of their care team having access to their wearable activity data. Health professionals often do not have access to these data sets without the patient bringing in their device to a consultation. This presents problems for patients who are living in rural and remote areas and may increase the need for face-to-face appointments. In our study, the information from the wearable devices was able to be accessed remotely, which enabled more individualized feedback to cohort 3.

Our study showed that real-time monitoring of symptoms and treatment-related side effects can be reported through remote systems and the use of these systems is acceptable, which is consistent with previous research [22-23]. Furthermore, in addition to increased patient HRQoL, a recent randomized controlled trial reported that there may be additional benefits to patients’ overall survival for those who monitor their symptoms longitudinally [24].

PRO completion rate for cohorts 1 and 2 was lower than for cohort 3. Only two automated attempts to encourage participants to provide follow-up PRO data were made to each cohort during the study. Further individualized contact may be needed to collect such data when using remote models of care.

The usefulness of educational material is likely to be dependent on participants’ stages of disease, cancer treatment, and trajectory. Tailoring of educational content in this Web portal was insufficient to account for individual needs and stages of treatment, recovery, and health literacy. This finding is supported by previous reviews in various populations that indicate digital health interventions need to focus on increasing personal relevance of content [25-27]. Further research is needed to determine which type of educational content is most appropriate and useful at various time points in a patients’ care pathway.

The inclusion of qualitative interviews provided important insights into participant perspectives of the intervention. These data have been helpful in conceptualizing changes to the Web portal, the intervention, and future research studies. Codesigned health systems have been shown to increase functionality, specificity, and uptake [28].

Reduced physical activity levels during cancer treatment can lead to increased symptom burden and, consequently, reduced quality of life. There is no single solution to facilitate positive behavior change across a population of people with cancer in active treatment; however, the innovative use of technology may benefit a proportion of the population.

### Limitations

More participants in cohorts 2 and 3 were receiving chemotherapy during the study period, and cohort 1 participants had been off treatment longer than the others. Since these differences were not accounted for in the data provided, it is unknown what impact they had on the findings.

The study also had small group sizes and heterogeneous cancer diagnoses of the participants. Broad inclusion criteria were appropriate for this feasibility study to increase generalizability to the larger population of cancer survivors. However, future studies may need to consider the specific requirements of different cancer diagnoses and stages of disease in order to provide appropriately tailored interventions effectively.

All participants had access to a mobile phone, which may define them as different from the general cancer population and could result in overestimation of the feasibility and acceptability of the program. However, only 6% of screened participants were excluded due to lack of a mobile phone, suggesting that the study group was representative of the general cancer population in regard to the use of mobile phones.

The utility of Web portals for clinicians and clinician-patient relationships is an important benefit of such systems. This study did not include data review or interactions with medical specialists and was limited to interactions with an exercise physiologist.

### Future Iterations

Development of an automated alert algorithm focused on a combination of PRO measures, symptom tracking, and activity monitor data could improve functionality of the Web portal. For example, if pain above a set value for a set number of days were reported, this would trigger a clinical message to the patient’s care team for investigation. Alerts and flagging mechanisms triggering clinician intervention for patients with cancer to report their symptoms have been shown to be effective in several studies; however, none of these studies included integration of wearable activity monitor data [29-32].

The Web portal was not fully integrated into care pathways and the hospital electronic medical record (EMR) as this integration was cost- and time-prohibitive when developing this study. Integration of data into the EMR is a potential area of future development for this Web portal. EMR integration provides opportunity for multiple members of the patient’s care team to provide remote monitoring and support. Integration of remote tracking data into the EMR also increases clinical metrics available to clinicians to inform decision making and referral practices. For example, a patient reporting cancer-related fatigue corresponding with low physical activity levels could be appropriately referred to a local exercise oncology professional for individualized exercise counseling and prescription.

Further studies may also consider the inclusion of health economic data. Health professionals are typically time poor, and although this study indicated that weekly time spent for each participant receiving coaching was minimal, future research should report this in greater detail as well as the travel time saved by patients.

Our findings from this prospective cohort study indicate it is a feasible digital health tool for people with a history of cancer. Tailored messaging is needed to maximize engagement in this population. It is anticipated that the results of this pilot will inform the design of an adequately powered randomized controlled trial assessing the efficacy of this intervention.

## Abbreviations

CBI-B: Cancer Behavior Inventory–Brief Version
EMR: electronic medical record
ESAS: Edmonton Symptom Assessment Scale
FACT-G: Functional Assessment of Cancer Therapy–General
HRQoL: health-related quality-of-life
IQR: interquartile range
PRO: patient-reported outcome
